# Kisspeptins Modulate the Biology of Multiple Populations of Gonadotropin-Releasing Hormone Neurons during Embryogenesis and Adulthood in Zebrafish (*Danio rerio*)

**DOI:** 10.1371/journal.pone.0104330

**Published:** 2014-08-05

**Authors:** Yali Zhao, Meng-Chin A. Lin, Allan Mock, Ming Yang, Nancy L. Wayne

**Affiliations:** 1 Department of Physiology, David Geffen School of Medicine at University of California Los Angeles, Los Angeles, California, United States of America; 2 School of Environmental and Chemical Engineering, Shanghai University, Shanghai, China; University of Otago, New Zealand

## Abstract

Kisspeptin1 (product of the *Kiss1* gene) is the key neuropeptide that gates puberty and maintains fertility by regulating the gonadotropin-releasing hormone (GnRH) neuronal system in mammals. Inactivating mutations in *Kiss1* and the kisspeptin receptor (*GPR54/Kiss1r*) are associated with pubertal failure and infertility. *Kiss2*, a paralogous gene for *kiss1*, has been recently identified in several vertebrates including zebrafish. Using our transgenic zebrafish model system in which the GnRH3 promoter drives expression of emerald green fluorescent protein, we investigated the effects of kisspeptins on development of the GnRH neuronal system during embryogenesis and on electrical activity during adulthood. Quantitative PCR showed detectable levels of *kiss1* and *kiss2* mRNA by 1 day post fertilization, increasing throughout embryonic and larval development. Early treatment with Kiss1 or Kiss2 showed that both kisspeptins stimulated proliferation of trigeminal GnRH3 neurons located in the peripheral nervous system. However, only Kiss1, but not Kiss2, stimulated proliferation of terminal nerve and hypothalamic populations of GnRH3 neurons in the central nervous system. Immunohistochemical analysis of synaptic vesicle protein 2 suggested that Kiss1, but not Kiss2, increased synaptic contacts on the cell body and along the terminal nerve-GnRH3 neuronal processes during embryogenesis. In intact brain of adult zebrafish, whole-cell patch clamp recordings of GnRH3 neurons from the preoptic area and hypothalamus revealed opposite effects of Kiss1 and Kiss2 on spontaneous action potential firing frequency and membrane potential. Kiss1 increased spike frequency and depolarized membrane potential, whereas Kiss2 suppressed spike frequency and hyperpolarized membrane potential. We conclude that in zebrafish, Kiss1 is the primary stimulator of GnRH3 neuronal development in the embryo and an activator of stimulating hypophysiotropic neuron activities in the adult, while Kiss2 plays an additional role in stimulating embryonic development of the trigeminal neuronal population, but is an RFamide that inhibits electrical activity of hypophysiotropic GnRH3 neurons in the adult.

## Introduction

KISS1 and its receptor play major roles controlling the reproductive system in vertebrates [1], [2], [3], [4], [5]. In humans, mutations of the KISS1 receptor (GPR54, more recently termed KISS1R) [4], [6] and a recently described inactivating *KISS1* mutation [7] lead to hypogonadotropic hypogonadism. In mice, *Kiss1* and *Kiss1r* genetic knockdown models also show significant deficits in pubertal development and reproductive functions [1], [4], [8]. In mammals, the *Kiss1r* is expressed widely in the brain, including on GnRH1 neurons that form the final common pathway regulating the pituitary-gonadal axis [9], [10], [11]. Studies in mice indicate that kisspeptin can stimulate GnRH1 neuronal activities through both direct and indirect actions involving synaptic transmission [12], [13].

Two forms of the *kiss* gene (*kiss1, kiss2*) and the *kiss* receptor gene (*kiss1r, kiss2r*) have been identified in the nervous system of teleost fishes, including the species used in the present study – zebrafish (*Danio rerio*) [14], . Studies in adult zebrafish showed that Kiss1 neurons are located in the habenula, while Kiss2 neurons are located in the posterior tuberal nucleus and the dorsal and ventral hypothalamus [18], [19]. Kiss1 neurons projected to the interpeduncular and raphe nuclei where the Kiss1r is expressed; while Kiss2 neurons projected to the subpallium, the preoptic area (POA), the thalamus, the ventral and caudal hypothalamus, and the mesencephalon where the Kiss2r is expressed [18], [20]. The ventral forebrain (including POA and hypothalamus) is rich in GnRH3 neurons that regulate reproduction in zebrafish, and it was found that Kiss2 neural processes were in close apposition to GnRH3 neurons in this region [18]. The localization of Kiss1 and Kiss 2 neurons suggests that in adult zebrafish, Kiss2 neurons are the primary regulators of the brain-pituitary-gonadal axis, while Kiss1 neurons serve other functions (such as regulating the serotonergic system) [19], [20].

Vertebrates express 2–3 forms of GnRH, with distinct genes, locations and functions [21]. Zebrafish is unusual for teleost fishes in that it expresses only two forms of GnRH. Its genome is missing an ortholog to mammalian GnRH1 [22], but expresses GnRH2 in midbrain tegmentum and GnRH3 in forebrain areas including the terminal nerve, ventral telencephalon, POA and hypothalamus [23], [24], [25], [26]. GnRH3 neurons located in ventral telencephalon, POA and hypothalamus of zebrafish have taken on a hypophysiotropic role attributed to GnRH1 neurons in other species [23], [25]. GnRH3 is also expressed in trigeminal ganglia of the peripheral nervous system of medaka and zebrafish embryos [26], [27], [28]. Presumably, these sensory neurons, as well as GnRH3 neurons located in the terminal nerve associated with the olfactory region, play neuromodulatory roles not directly related to reproduction [26], [29], [30].

Given that zebrafish *kiss1* and *kiss2* mRNAs are expressed during embryonic and larval development [16], we explored the relative actions of Kiss1 and Kiss2 on morphological development of multiple populations of GnRH3 neurons during embryogenesis. We also investigated the effects of Kiss1 and Kiss2 on the electrical activity of hypophysiotropic GnRH3 neurons within its intact neural network from excised adult brain. Much of this work was accomplished using a unique transgenic zebrafish model in which the GnRH3 promoter drives expression of a bright variant of green fluorescent protein (Emerald GFP or EMD; [30]). Because zebrafish embryos are transparent, neurons expressing the GnRH3:EMD transgene are readily identifiable in the live embryo. This animal model provides an outstanding opportunity to visualize GnRH3 neurons during embryogenesis in order to follow their development in the live embryo, as well as to identify these neurons for electrophysiological recording in the intact brain [30].

## Materials and Methods

### Animals

Brass GnRH3:EMD transgenic zebrafish were maintained in a zebrafish aquarium system on a 14L:10D photoperiod at 28°C. They were fed twice daily with flake food and live brine shrimp. Sexually mature males and females were maintained in separate tanks until the day before breeding. To control the breeding time, each male-female pair was set in one tank with a divider to separate them. The divider was removed shortly after lights on to allow timed breeding, and fertilized eggs were collected. Embryos were maintained in a 28°C incubator. All embryos for a single set of experiments were from the same parents.

### Ethics Statement

All procedures were carried out in accordance with and approved by the Animal Care and Use Committee of UCLA (protocol # 2003-101-32; approval period from February 22, 2013 through February 21, 2015).

### Treatments with Kiss1 and Kiss2

Biologically-active, 10-amino acid fragments of zebrafish Kiss1 (YNLNSFGLRY-NH_2_) and Kiss2 (FNYNPFGLRF-NH_2_) were synthesized by Bachem Inc. (Torrance, CA), based on the sequence described by Kitahashi and co-workers [16] and previously described by Zhao and Wayne (2012) [31]. Stock solutions of 800 µM Kiss1 (10) and 1 mM Kiss2 (10) were made in acidified albumin-saline (0.6 ml 12 N HCl, 1.0 g Fraction V bovine serum albumin, 8.5 g NaCl per 1.0 L water; T. Pak, personal communication), stored at −80°C, and then diluted in fish saline at a final concentration of 100 nM just prior to experimentation. Control solution was treated in the same way as the treatment solutions such that the final solutions throughout a given experiment contained the same amount of acidified albumin-saline [31].

Two sets of experiments were performed with kisspeptin treatments, confocal imaging of embryonic and adult GnRH neurons, and electrophysiology of adult hypophysiotropic GnRH3 neurons. For imaging studies, embryos either with the chorion intact or removed were incubated in 100 nM Kiss1 or Kiss2 solutions starting at 5 hours post-fertilization (hpf) until they were sacrificed, and then fixed with 4% paraformaldehyde (PFA) at various developmental stages for morphological analysis by confocal microscopy. A comparison between the presence and absence of the chorion made no difference to the outcome of kisspeptin treatments on GnRH3 neuron development (data not shown). This indicates that kisspeptin diffuses through the chorion efficiently. In the study on adult zebrafish, embryos were treated with kisspeptins starting at 5 hpf until 3 dpf, and then raised to adulthood in the zebrafish aquarium facility starting at 5 dpf. Adult brain imaging studies were performed at about 8 months of age. For electrophysiology experiments, following the protocol described by Zhao and Wayne (2012) [31], 100 nM Kiss1 or Kiss2 solutions were applied by a flow-through perfusion system at a speed of 200 µl/min.

### Quantitative PCR analysis of *kiss1* and *kiss2* gene expression in embryos and/or larvae

To test the early expression of *kiss1* and *kiss2*, embryos were collected daily from 1–7 days post fertilization (dpf) in four replicate experiments. Using RNeasy Plus Mini Kit (Qiagen, USA), total RNA was extracted from homogenates of 20–50 zebrafish embryos or larvae collected at different developmental stages. Reverse transcriptase reaction was performed from 0.5 µg of total RNA using the MultiScribe Reverse Transcriptase (Applied Biosystems, Foster City, CA). The following is the Reverse Transcriptase (RT) reaction mixture and procedure: 0.5 µg RNA, 10 µl of 2× RT buffer, 1 µl of 20× RT enzyme mixture and RNAase-free water to a final volume of 20 µl. The mixture was incubated at 37°C for 60 minutes and then heat inactivated at 95°C for 5 minutes.

Quantitative real-time PCR was conducted using the SYBR Green PCR Master Mix (Applied Biosystems, Foster City, CA) following the manufacturer's instructions in an Mx 3000P System (Stratagene, La Jolla, CA). The reaction was composed of the following: 1.5 µl of the RT reaction, 10 µl of 2× qPCR mixture with the appropriate forward and reverse primers to a final volume of 20 µl. The final concentrations of the primers in the qPCR reaction were 1.25 µM. The nucleotide sequences of the qPCR *kiss1* and *kiss2* primers were previously described by Kitahashi, and co-workers (2009) [16]. The PCR reactions were set as follows: 95°C for 10 minutes, followed by 40 cycles of 95°C for 15 seconds, and 60°C for 1 minute. The fluorescent signals were measured at the annealing/extension step. Melting curve analyses were performed to validate the specificity of PCR amplicons. *Elongation factor 1a (ef1a)* was used as an internal reference gene for each tested sample. Each sample was processed for quantitative PCR in triplicate. A cycle threshold (Ct)-based relative quantification with efficiency correction normalizing to *ef1a* was calculated by the 2^−ΔΔCt^ method. The relative quantity of *kiss1 or kiss2* gene expression was presented as the fold difference to that of 1 dpf embryos. The sequences of the primers used for quantitative RT-PCR are shown in Table 1. The PCR primer sets met specificity and efficiency requirements: dissociation curves produced single bands; replicon DNA sizes were as expected as determined by DNA gel electrophoresis; and the efficiencies for *ef1a*, *kiss1* and *kiss2* primer sets were 88%, 100%, and 108%, respectively, as determined by serial dilution experiments.

**Table 1:** The DNA sequences of primers used for quantitative RT-PCR.

Gene	DNA sequence of primers	Amplicon length (bp)
*ef1a*	Forward 5′-AAGACAACCCCAAGGCTCT -3′Reverse 5′-CCTTTGGAACGGTGTGATTGA-3′	255
*kiss1*	Forward 5′-ACAAGCTCCATACCTGCAAGTG-3′Reverse 5′-AATACTGAAAATGCCCAGAGGG-3′	131
*kiss2*	Forward 5′-GCCTATGCCAGACCCCAAA-3′Reverse 5′-TTTACTGCGTGCTAGTCGATGTTT-3′	155

### Immunohistochemistry of SV2

Synaptic vesicle protein 2 (SV2) is a marker of synaptic transmission, including in zebrafish [32], [33], [34], [35]. Whole mount immunochemical staining with SV2 antibody was performed as described previously [26]. Briefly, embryos (50 hpf) preincubated in control or kisspeptin solutions from 5–50 hpf were fixed in 4% PFA overnight at 4°C, permeabilized in ice cold acetone for 8 minutes, rinsed in 0.3% Tween solution in PBS, then blocked for 2 h at room temperature in the dark with a solution of 0.3% Tween and 10% goat serum (in PBS). The samples were incubated in 1∶200 SV2 antibody (mouse, monoclonal, purchased from Developmental Studies Hybridoma Bank, University of Iowa) in blocking solution overnight at 4°C. The next day, diluted secondary antibody (Alexa Fluor 594 anti-mouse, 1∶1000; Invitrogen, Carlsbad, CA) was applied overnight at 4°C. After completion of 5× rinses (10 min each), the whole embryos were mounted in 0.8% agarose for confocal microscopy imaging.

### Confocal microscopy

Images were acquired and analyzed with Fluoview software using an upright Olympus microscope (Olympus America Inc., Center Valley, PA). Three sets of experiments were performed: 1) analyze neuron number from the different GnRH3 neuronal populations to assess the effects of kisspeptins on early embryonic development of the GnRH3 neural circuit (18–25 hpf); 2) analyze the number of synaptic boutons on the cell bodies and along the neuronal processes of the bilateral clusters of TN-GnRH3 neurons to evaluate the effects of kisspeptins on potential synapse formation during embryogenesis (50 hpf) by means of whole mount immunohistochemical staining with SV2 antibody; 3) analyze the number of hypothalamic GnRH3 neurons from adult zebrafish that were treated with kisspeptins as embryos to determine if early changes continued into adulthood. Embryos were mounted in 0.8% agarose in two positions, lateral-side up (to best visualize the trigeminal population of GnRH3 neurons) or ventral-side up (to best visualize the forebrain populations of GnRH3 neurons), then viewed and imaged using water immersion 5×, 20× or 40× objectives (Olympus America Inc., Center Valley, PA). Z-stacks of images were taken at the interval of 0.5 µm.

To count the number of boutons, we overlaid the projection images of both green-488 (EMD) and red-594 (SV2-ir) channels of bilateral TN-GnRH3 neuron clusters and their processes taken with 40× water objective with 3× digital zoom, counted the yellow (co-localization) spots (>0.1 µm) on the cell bodies and along the neuronal processes projecting from the bilateral clusters, section by section through the projection stacks [36],[37]. We then verified the bouton numbers by re-analyzing the images viewed in 3D. Projection images were used for display and analysis. Analysis of numbers of cell bodies and boutons was performed by one of the authors (MAL) who was blind to the treatments. This study was conducted in embryos at 50 hpf because SV2 is strongly expressed in the forebrain at this time and not at the earlier developmental time of 25 hpf [26]. Because the TN-GnRH3 neuronal processes from a single cluster wrap together, it was not possible to identify which process projects from which neuron – we could only identify processes projecting from a TN-GnRH3 cluster. Therefore, the total number of synaptic boutons was normalized to the total number of GnRH3 neurons in the bilateral terminal nerve clusters.

To assess whether or not embryonic treatment with kisspeptins had long lasting effects into adulthood, embryos were treated 100 nM Kiss1, 100 nM Kiss2, or control solution (5 hpf- 3 dpf) and maintained in a zebrafish aquarium system for eight months until sacrifice. Adult intact brains were removed and fixed in 4% PFA/10% sucrose in PBS at 4°C overnight. After fixation they were washed three times with 20% sucrose in PBS and were incubated at 4°C overnight. The brains then were left in the mixture of 20% sucrose and OCT compound (Tissue-Tek) (1∶2) again at 4°C overnight. The next day, the brains were sliced horizontally (50 µm thickness) using a cryostat (Leica CM1850) at −20°C. Fluoview software was used to image and to count the number of GnRH3-EMD neurons in hypothalamus using confocal imaging (1 µm optical slices, 10×). Analysis of numbers of cell bodies was performed by one of the authors (MAL) and another researcher (MF) who were blind to the treatments.

### Electrophysiology

Whole-cell patch electrophysiology was conducted as previously described [38]. Briefly, adult zebrafish (3–6 months of age) were anesthetized by immersion in MS- 222 (150 mg/L) and decapitated. The entire brain was carefully removed from the skull and then glued ventral-side up to a glass coverslip at the bottom of a flow-through recording chamber (P1; Warner Instrument Corp., Hamden, CT). The meninges were gently peeled away to expose the POA and hypothalamus. EMD labeled GnRH3 neurons in POA and hypothalamus were visualized under an upright microscope (BX50W, Olympus, Melville, NY, USA). Whole-cell recordings of membrane potential (Vm) and spontaneous action potentials were recorded in current-clamp mode. Data was acquired by PowerLab instrumentation and software (ADInstruments Inc., Colorado Springs, CO, USA), and analyzed by AxoGraph software (Axon Instruments, Foster City, CA, USA). Data were collected if interspike Vm was at least −40 mV. Following a stable baseline recording, 100 nM of Kiss1 or Kiss2 in fish saline was applied through the perfusion system for 5 min (it takes about 4 min to reach final concentration in the recording chamber with a perfusion rate of 200 µl/min). Normal fish saline was used for the baseline period and washout of the drugs. Aerated solutions were perfused continuously through the recording chamber. One neuron was recorded per animal. To quantify the effects of kisspeptins, the membrane potential and firing frequency during the last minute of the baseline and treatment periods were measured and analyzed.

### Data analysis

Values are shown as mean ± SEM. Data were analyzed using Prism 6 (GraphPad Software, Inc., San Diego, CA, USA). Statistical significances among experimental groups in the imaging studies were determined by one-way ANOVA, followed by the Tukey's multiple-comparison test. Paired t-test was used for analysis of electrophysiological data. Differences were considered significant if *p*<0.05.

## Results

### *Kiss1* and *kiss2* gene expression during embryonic and larval development

**Figure 1:**
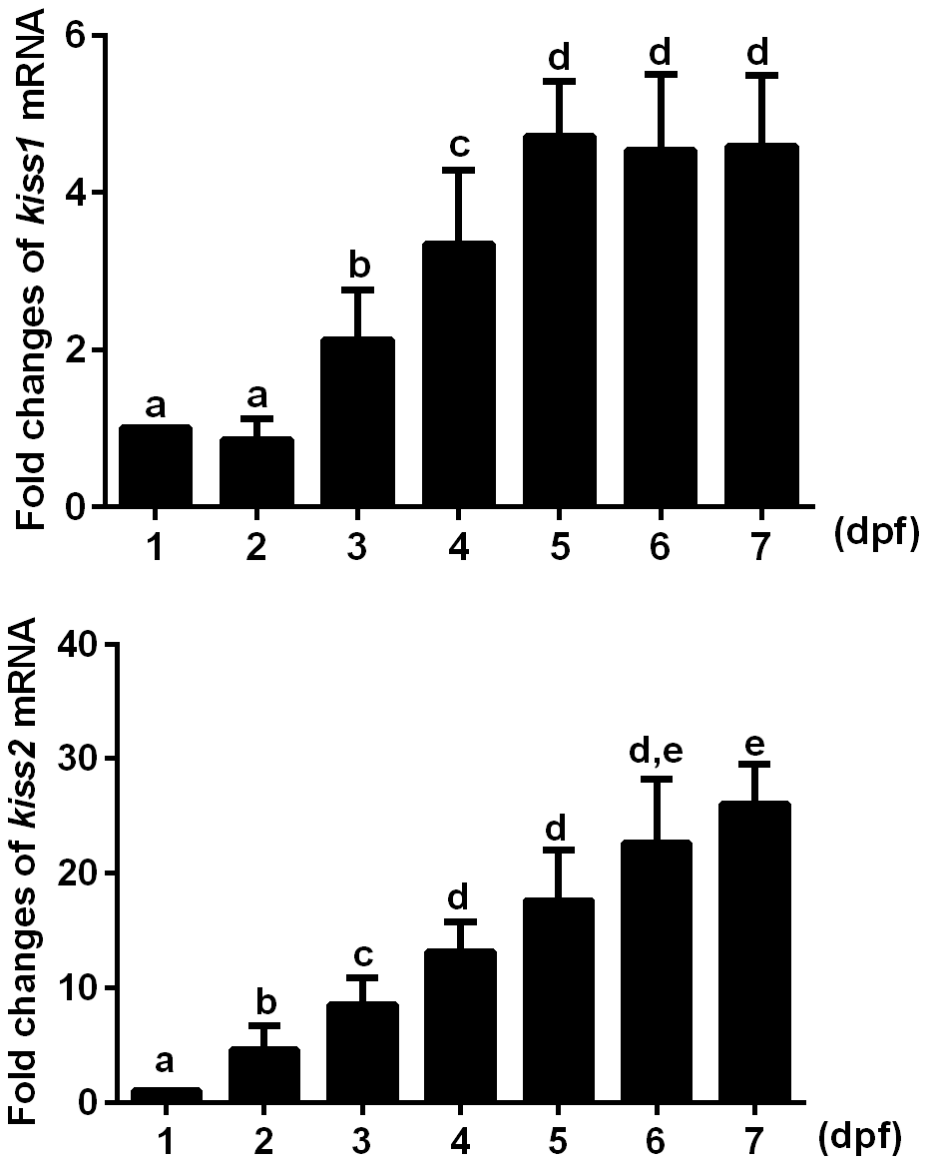
Levels of *kiss1*and *kiss2* mRNAs in embryonic and larval zebrafish (1–7 days post-fertilization (dpf). Quantitative RT-PCR values were normalized against *elongation factor 1a* and represent fold changes of *kiss1* mRNA (top panel) and *kiss2* mRNA (bottom panel) relative to that of 1 dpf embryos (n = 4 replicate experiments). Different letters denote significant differences between groups (at least P<0.05).

Using quantitative RT-PCR, we tested the dynamic changes of both *kiss1* and *kiss2* gene expression from 1–7 dpf (Figure 1). Both *kiss1* and *kiss2* mRNAs were detectable at 1 dpf and then increased during the first week of life, similar to the results reported by Kitahashi and colleagues (2009) [16]. However, *kiss2* mRNA increased more dramatically than *kiss1* mRNA between 1 and 7 dpf (*kiss2*: 26.04±1.74 fold increase; *kiss1*: 4.59±0.45 fold increase).

### Effects of Kiss1 and Kiss2 on proliferation of trigeminal GnRH3 neurons during embryogenesis

**Figure 2:**
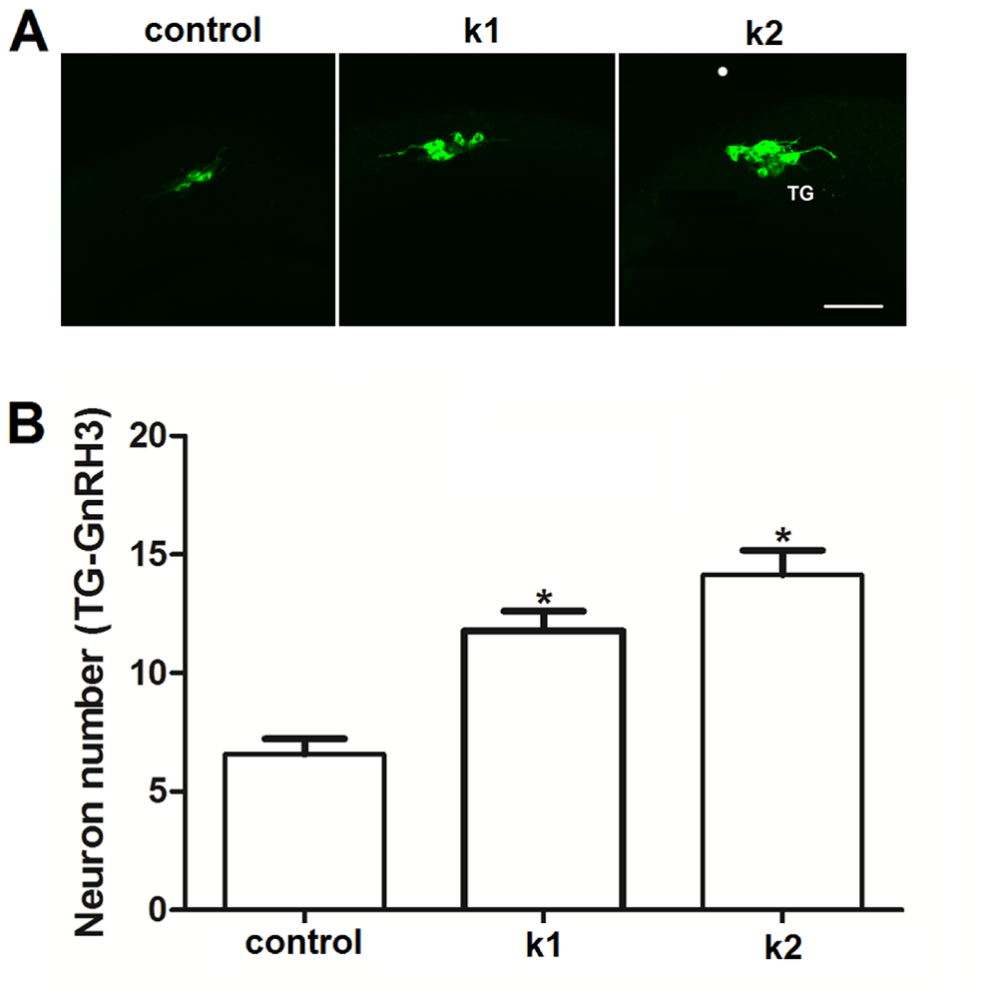
Kiss1 and Kiss2 stimulate proliferation of trigeminal GnRH3 neurons. (A) Confocal images of lateral view of trigeminal (TG) GnRH3:EMD neurons at 18 h post fertilization (hpf). Scale bar, 50 µm. (B) Both Kiss1 (k1) and Kiss2 (k2) increased the number of TG-GnRH3:EMD neurons compared to controls at 18 hpf (at least n = 7 embryos per group). *: *p*<0.05 compared to controls.

Our previous work [26] revealed that trigeminal GnRH3 neurons in zebrafish was one of the earliest of the GnRH3 populations to emerge in this transgenic model system, starting around 15 hpf. Using confocal microscopy we counted the number of GnRH3:EMD expressing neurons in this population at 18 hpf after exposure to control solution, 100 nM Kiss1, or 100 nM Kiss2 (treatment duration: 5–18 hpf). Figure 2 shows that both Kiss1 and Kiss2 significantly increased the number of trigeminal GnRH3 neurons compared to control treatment (control, 6.57±0.65, n = 7; Kiss1, 11.78±0.83, n = 9; Kiss2, 14±1.03, n = 7). There was no significant difference in neuron numbers between the Kiss1 and Kiss2 treatment groups.

### Effects of Kiss1 and Kiss2 on proliferation of forebrain GnRH3 neurons during embryogenesis

**Figure 3:**
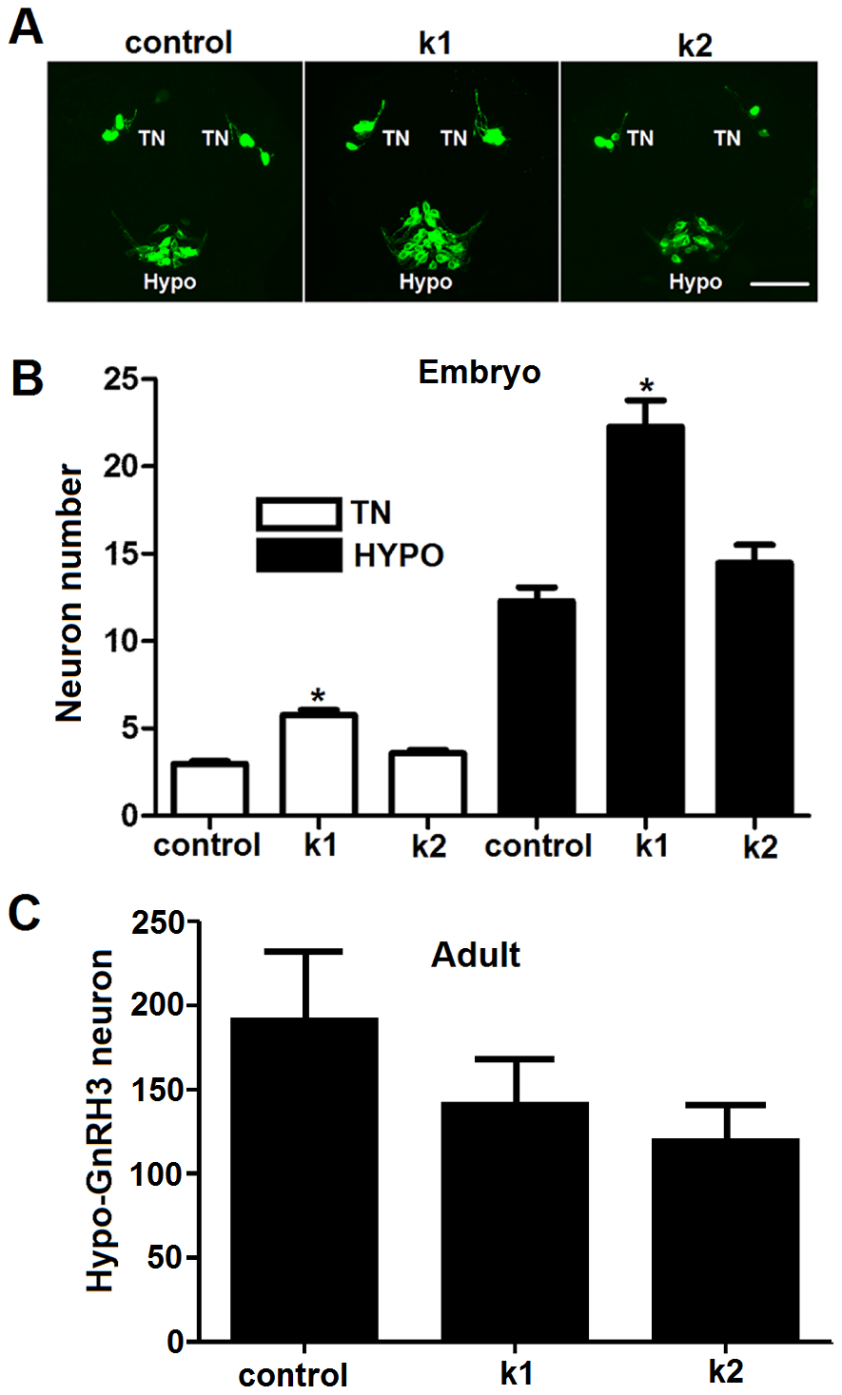
Kiss1 but not Kiss2 stimulates proliferation of terminal nerve and hypothalamic GnRH3 neurons in embryos. (A) Confocal images of ventral view showing the expression of TN-GnRH3:EMD neurons (TN) and hypothalamic GnRH3:EMD neurons (HYPO) at 25 hpf of a representative control embryo, and embryos treated with Kiss1 (k1) and Kiss2 (k2). Scale bar, A: 50 µm. (B) Summary data of the number of neurons expressed at 25 hpf in TN and HYPO (at least n = 14 embryos per group). Kiss1, but not Kiss2, significantly increased GnRH3 neuron numbers in both terminal nerve and hypothalamus. *: *p*<0.05 compared to controls. (C) Summary data of the number of hypothalamic GnRH3:EMD neurons in adult brains of zebrafish treated as embryos with 100 µM Kiss1 (n = 5), 100 µM Kiss2 (n = 4) or control solution (n = 5) (treatment duration: 5 hpf - 3 dpf).

Our previous study showed that multiple populations of GnRH3 neurons emerged in this transgenic zebrafish model by 30 hpf [26]. In forebrain, both terminal nerve (TN) and hypothalamic populations appeared around 20 hpf. To test if kisspeptins affect early GnRH3 neural development in the brain, we fixed the embryos with 4% PFA at 25 hpf after exposure to 100 nM Kiss1 and Kiss2 (treatment duration: 5–25 hpf). The neuron numbers of these two populations were counted and analyzed using confocal imaging of embryos in a ventral side up position. Results are shown in Figure 3. The data indicate that Kiss1, but not Kiss2, significantly increased the number of TN and hypothalamic GnRH3 neurons at this early stage of embryonic development. The increase in number of hypothalamic GnRH3 neurons in response to Kiss1 (treatment duration: 5 hpf – 3 dpf) did not extend into adulthood (Fig. 3), indicating that effects of treatment with kisspeptin on GnRH3 neuron number during embryogenesis are transient.

### Effects of Kiss1 and Kiss2 on the number of boutons on neural processes of terminal nerve GnRH3 neurons

**Figure 4:**
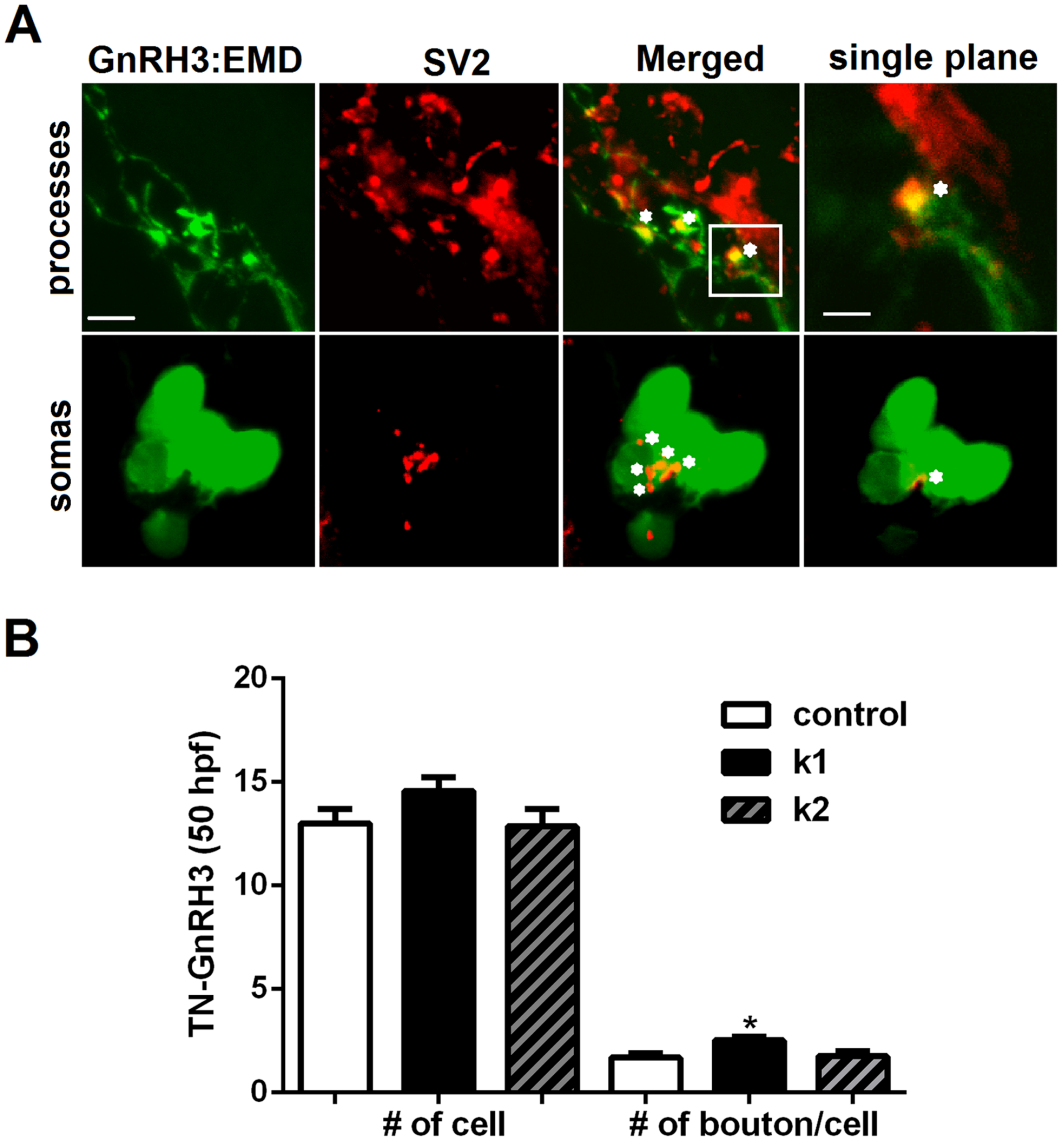
Effects of Kiss1 and Kiss2 treatments on bouton formation on terminal nerve (TN)-GnRH3 neurons. (A) Representative images of a TN-GnRH3:EMD neuron cluster in a 50 hpf embryo from the Control group, showing synaptic boutons in the neuronal processes (upper panels) and somas (lower panels) labeled with antibody to the presynaptic marker SV2. *Far left panels*: EMD expression in GnRH3 neurons (green); *Middle left panels*: Immunoreactive SV2 (red); *Middle right panels*: merged images of EMD and SV2 showing synaptic boutons in yellow; *Far right panels*: upper image: single plane (1 µm optical slice) with higher magnification of the boxed area in the upper middle right panel showing the synaptic contacts in the neuronal processes; lower image: single plane (1 µm optical slice) showing a synaptic bouton on the soma. Each asterisk labels a nearby bouton. (B) Total number of TN-GnRH3:EMD neurons in the bilateral clusters (left), and number of boutons normalized to the total number of neurons in the clusters (soma and processes; right). Kiss1 and Kiss2 had no effect on the number of TN-GnRH3 neurons at 50 hpf. However, Kiss1, but not Kiss2, significantly increased the number of boutons per TN-GnRH3 neuron (n = 7 embryos per group). This increase was seen in both cell bodies and neural projections. *: *p*<0.05 compared to controls. Scale bars: 5 µm for all images, except the one in the upper far right panel where it is 2 µm.

Our earlier work showed that TN-GnRH3 neurons express synaptic vesicle protein (SV2) on their neural processes during development, suggesting that these neurons are capable of synaptic communication during embryogenesis [38]. In the present study, we investigated the effects of Kiss1 and Kiss2 on the formation of synaptic contacts by analyzing the number of boutons on cell bodies and neural processes of TN-GnRH3 neurons in embryos that were 50 hpf (treatment duration: 5–50 hpf). Figure 4 shows that compared to the untreated control group, embryos treated with Kiss1 developed significantly more boutons on TN-GnRH3 neurons. This stimulatory effect was observed for both cell bodies and neural processes (total boutons normalized to the total number of TN-GnRH3 neurons shown in Fig. 4. Kiss2 had no effect.

### Effects of Kiss1 and Kiss2 on electrical activity of hypophysiotropic GnRH3 neurons in the adult intact brain

**Figure 5:**
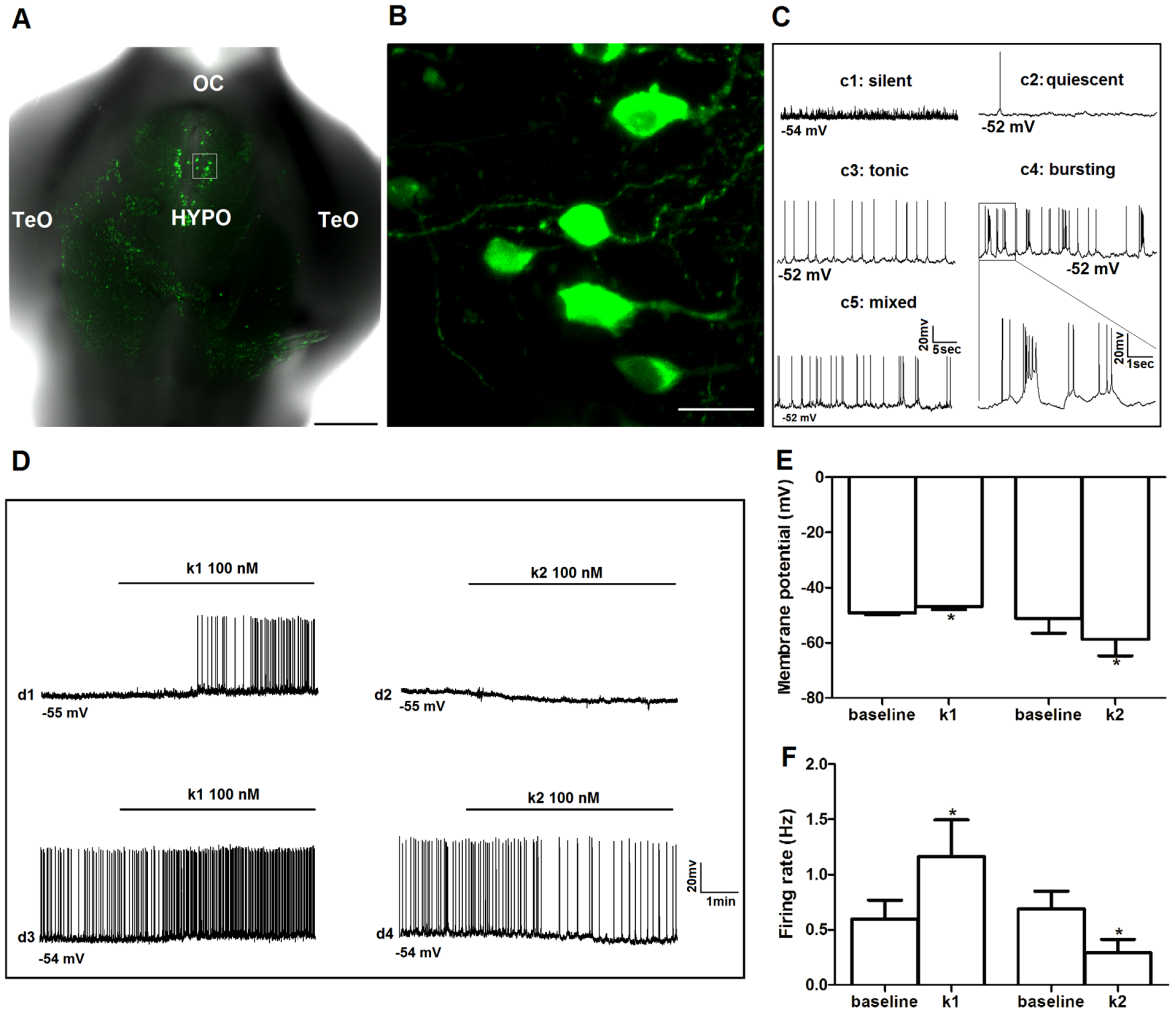
Effects of Kiss1 and Kiss2 on electrical activity of hypophysiotropic GnRH3:EMD neurons in adult zebrafish brain. (A) Low power confocal image of ventral view of excised, intact brain showing GnRH3:EMD neurons expressed in hypothalamus. OC: optic chiasm; HYPO: hypothalamus; TeO: optic tectum. Scale bar, 200 µm. (B) High magnification confocal image of the white boxed region in (A). Scale bar, 10 µm. (C) Sample electrophysiology traces of different patterns of spontaneous action potential firing from GnRH3:EMD neurons located in the preoptic area and hypothalamus of the intact adult brain: silent (c1); quiescent (c2); tonic (c3); bursting (c4); mixed tonic and bursting (c5). The boxed area in c4 is expanded to show details of the firing pattern. (D) Sample traces from four neurons showing the responses to Kiss1 (k1: d1 and d3) and Kiss2 (k2: d2 and d4), with 2 min baseline and 5 min treatment period. d1 and d2: silent neurons; d3 and d4: active neurons. Summary of the effects of k1 and k2 treatments on membrane potential (E) and spike frequency (F). *: *p*<0.05 compared to baseline values. At least n = 6 neurons for each treatment group.

The functions of GnRH3 neurons are dependent upon their locations in zebrafish [24]. Like GnRH1 in mammals, hypophysiotropic GnRH3 neurons that regulate the pituitary-gonadal axis are located in the POA and hypothalamus [5]. In zebrafish, many neurons in these forebrain populations are located near the ventral surface of the brain and are accessible for electrophysiology in the excised, intact preparation in which most neural circuits are preserved [38] (Fig. 5A,B). We conducted whole-cell patch electrophysiology to study the effects of Kiss1 and Kiss2 on membrane potential and spontaneous action potential firing of GnRH3 neurons in the POA and hypothalamus. Out of 25 neurons recorded in this study, 12 (48%) of them were silent or quiescent (firing rate in 5 min: <0.05 Hz) and 13 (52%) were active (firing rate in 5 min: 0.23–2.6 Hz) during the baseline-recording period. There were three firing patterns recorded in the active neurons: bursting (25%), tonic (42%) and mixed (33%). Example recording traces are shown in Figure 5C. As expected, the silent/quiescent neurons were more hyperpolarized than the active ones (silent/quiescent: −57.14±1.84; active: −50.45±2.84; P<0.05). There were no sex differences in Vm or spike frequency in any of the groups.

Figure 5D–F shows that Kiss1 and Kiss2 had opposite effects on membrane potential and spike frequency. First, in active neurons Kiss1 caused membrane potential to depolarize (baseline: −49.17±0.70 mV; Kiss1: −46.83±1.20 mV; n = 6), while Kiss2 caused it to hyperpolarize (baseline: −51.17±5.39 mV; Kiss2: −58.67±6.06; n = 6). There was a similar trend of changes observed for the silent/quiescent neurons (Kiss1, n = 8; Kiss2, n = 5), but the differences were not statistically significant (data not shown). Second, in active neurons Kiss1 increased spike frequency (from 0.59±0.17 to 1.16±0.33 Hz), but Kiss2 decreased spike frequency (from 0.69±0.16 to 0.29±0.12 Hz). Additionally, 5 out of 8 silent/quiescent neurons treated with Kiss1 became active following treatment; none of the 5 silent/quiescent neurons treated with Kiss2 showed a change in their electrical activity in response to treatment.

## Discussion

We show that *kiss1* and *kiss2* genes are expressed by 1 dpf, and their mRNA levels increase progressively during embryonic and larval development in zebrafish. These findings corroborate earlier work in zebrafish showing increased *kiss1* and *kiss2* mRNA expression between 1–7 dpf, with peak levels achieved just prior to or during the time of puberty [16]. Expression of *kiss1/2* during zebrafish embryogenesis is similar to what was observed in cultured embryonic brain explants from mice in which *Kiss1* was also expressed early in development [39]. In contrast, recent work in embryonic medaka showed that both *kiss1* and *kiss2* mRNA expression peak at somite stage 19 or 1 dpf before declining to low levels by stage 26 or 2 dpf. The expression of both *kiss* mRNAs stayed low through 8 dpf [40]. This is a very different temporal expression profile than seen in zebrafish embryos/larvae. This diversity of early expression profiles across different species (even between different fish species) suggests that the kisspeptin system has varied potentials for influencing embryonic development. Hodne and co-workers demonstrated that kisspeptin signaling in medaka fish was crucial for embryonic survival and morphogenesis [40]. Notably, anatomical characterization of kisspeptins and their receptors have not yet been described in embryonic medaka and zebrafish.

Both Kiss1 and Kiss2 stimulated the number of GnRH3 neurons in the developing trigeminal neuron population. This was the only effect of Kiss2 observed on any aspect of GnRH3 neuron biology during early development that we investigated. The trigeminal population of GnRH3 neurons is unusual because it is located in the peripheral nervous system. Trigeminal neurons are part of the sensory system and, in part, mediate the effects of mechanosensory stimuli on escape behavior in zebrafish [41]. Although the existence of GnRH3 neurons in the trigeminal ganglion has been previously described [26], [27], [28], they have no known function to date. Like the terminal nerve population of GnRH3 neurons, it likely plays a neuromodulatory role and is not directly involved in regulating the pituitary-gonadal axis [26], [29], [42]. Kiss1, but not Kiss2, also increased the number of GnRH3 neurons in the developing terminal nerve and hypothalamic populations. This result is similar to findings using cultured embryonic brain explants from mice, showing KISS1 stimulates growth and development of GnRH1 neurons [43]. Altogether, this suggests that Kiss1 has the potential for promoting development of multiple GnRH3 neuron populations during embryogenesis. The increase in hypothalamic GnRH3 neuron number in response to brief treatment with Kiss1 did not persist into adulthood. Perhaps longer treatment would have had more lasting effects. Nevertheless, changes in GnRH neuronal development have been shown to play an important role in other aspects of brain and retinal development in zebrafish embryos [44], [45].

Recent work from our laboratory showed the expression of SV2 on GnRH3 neurons during early embryogenesis in zebrafish, indicating the potential for synaptic transmission while the GnRH3 neural network was still developing [26], [38]. Here we sought to determine if kisspeptin could modulate synaptic communication of GnRH3 neurons during embryogenesis. We focused on the terminal nerve population because their neural processes are readily identifiable during embryogenesis. The existence/formation of boutons has been described during embryonic/larval development of the zebrafish nervous system [46], [47]. Further, SV2 is expressed on synaptic vesicles and is a widely used marker of synaptic transmission, including in the zebrafish nervous system [32], [33], [34], [35], [48], [49], [50]. Punctate expression of SV2 along axons has been verified as a method of identifying synaptic boutons [36], [37]. In the present study, Kiss1 (but not Kiss2) stimulated the number of boutons on TN-GnRH3 cell bodies and neural processes that co-express SV2 during embryogenesis. This is the first demonstration that kisspeptin has the potential to modulate synaptic transmission during embryogenesis in any system.

Recent work showed that Kiss1, but not Kiss2, could stimulate spike frequency of TN-GnRH3 neurons in the zebrafish embryo – but only in those neurons that showed a mature tonic pattern of action potential firing. TN-GnRH3 neurons that showed the more immature bursting pattern of action potential firing were unresponsive to the stimulatory actions of Kiss1 [26]. Earlier studies showed that as zebrafish embryos mature, the TN-GnRH3 neurons develop a pattern of action potential firing that is more typical of adult TN-GnRH3 neurons [30] – a steady and reliable tonic pattern of firing [51], [52]. However, adult hypophysiotropic neurons in mammals and fish show a variety of different patterns of action potential firing, including both tonic and bursting patterns of firing [53], [54], [55], [56], [57]. In the present study, Kiss1 (but not Kiss2) stimulated electrical activity of POA and hypothalamic GnRH3 neurons by depolarizing membrane potential and increasing spike frequency – regardless of the pattern of action potential firing. In contrast, Kiss2 inhibited these neurons by hyperpolarizing membrane potential and decreasing spike frequency. This finding suggests that Kiss1, but not Kiss2, stimulates spontaneous action potential firing of hypophysiotropic GnRH neurons, as it does in mammals.

The physiology data in the present study is at odds with anatomical data showing localization of mRNAs for *kiss1*, *kiss2*, and their receptors in adult zebrafish. The anatomical data suggests that Kiss2, and not Kiss1, is the primary regulator of the brain-pituitary-gonadal axis in zebrafish [16], [18], [20]. Further, a functional study showed that treatment with Kiss2 but not Kiss1 increased FSH-β and LH-β mRNA levels in the pituitary glands of adult zebrafish [16]. As the whole brain preparation with intact neural circuits was used in the present study, it is possible that the kisspeptin treatments are modulating GnRH3 neurons through both direct and indirect pathways involving complex neural relays that culminate in an overall response of Kiss1 being stimulatory and Kiss2 being inhibitory to the hypophysiotropic GnRH3 neurons. Studies in mice showed that KISS1 stimulated hypophysiotropic GnRH neurons through both indirect and direct pathways [12], [13]. Likewise, our work in medaka showed that Kiss1 stimulated terminal nerve-GnRH3 neurons through an indirect pathway involving synaptic transmission [31]. Alternatively, it is possible that Kiss2 can act as an inhibitory RFamide. Table 2 shows multiple sequence alignment (ClustalW) of the amino acid sequences of zebrafish Kiss1, Kiss2, gonadotropin inhibitory hormone 1 (GnIH1), GnIH2, GnIH3, and two RFamide-related peptides that inhibit TN-GnRH3 pacemaker activity in dwarf gourami fish [16], [58], [59]. The amino terminus of Kiss2 is homologous to that of GnIH1 and RFRP1, all ending in Leucine-Arginine-Phenylalanine. This is not the case with Kiss1 [16].

**Table 2:** Multiple sequence alignment of neuropeptides.

Neuropeptide	Sequence alignment
Zebrafish Kiss1	-----------------------------YNLN--------------------------------SFQ**LRY**
Zebrafish Kiss2	-----------------------------FNYN---------------------------------PFG**LRF**
Zebrafish GnIH1	SLEIQDFTLNVAPTSGGASSPTILRLHPIIPKPAHLHANLP**LRF**
Zebrafish GnIH2	----------------------------APKST-------------------------------INLP**QRF**
Zebrafish GnIH3	--------------------------SGTGPS------------------------------ATLP**QRF**
Medaka RFRP1	SLDLESFNIRVTPTSSKLNLPTIKLYPPTAKPLHMHANMP**LRF**
Medaka RFRP2	---------------------------VSNSS-------------------------------PNMP**QRF**

Overall, our findings that Kiss1 is the dominant stimulator of GnRH3-neuron morphological (present study) and electrophysiological development [26] in the embryo, as well as the primary stimulator of GnRH3 neuron electrical activity in the adult zebrafish does not support the current model that Kiss2 plays the dominant role in reproductive function in zebrafish. As indicated earlier, work in different species of fish suggested that Kiss2 is the dominant activator of the hypothalamo-pituitary-gonadal axis, while Kiss1 has the potential to act as a weak modulator of reproduction. The present study in zebrafish using different experimental approaches reveals an alternative model in which Kiss1 is the dominant regulator of GnRH3 neuronal functions in the embryo and adult, while Kiss2 plays a more selective role. Work in medaka fish showed that Kiss1 neurons, but not Kiss2 neurons, respond to estrogen treatment and express estrogen receptor-alpha – suggesting a primary role for Kiss1 in regulating reproduction in medaka [60], similar to the present findings in zebrafish. Recently the analysis of early embryogenesis with transgenic knockout phenotypes of *kiss1/kiss1r* and *kiss2/kiss2r* in medaka indicated that *kiss1* and both *kiss1r* and *kiss2r* play critical roles in neurulation and embryonic development, but not *kiss2* [40]. Work investigating the functional significance of kisspeptins in morone fishes demonstrated that the actions of Kiss1 and Kiss2 were gonadal-stage dependent. Specifically, during the prepubertal stage, Kiss2 was more potent than Kiss1 in elevating plasma luteinizing hormone levels and up-regulated *gnrh1* and *gpr54-2* expression. However, during gonadal recrudescence, Kiss1 was more potent than Kiss2 in elevating plasma luteinizing hormone levels; while Kiss2 down-regulated *gnrh1* and *gpr-54-2* expression [61]. These findings suggest that effects of kisspeptins on GnRH neurons are at least partly dependent on hormonal milieu. Recent work in mouse embryos showed that kisspeptin receptor expression on GnRH1 neurons began at embryonic day 13.5 and that kisspeptin neurons make contact with GnRH1 neurons *in utero* [62], supporting our findings in the present study.

In summary, the present morphological findings in embryos show that kisspeptins modulate the development of multiple populations of GnRH3 neurons, suggesting it may play a role as a neurotrophic factor and possibly coordinate maturation of this neural network. The electrophysiology data in adult zebrafish suggests that both Kiss1 and Kiss2 regulate the activities of hypophysiotropic GnRH3 neurons. Kiss1 is an activator, while Kiss2 is an inhibitory RFamide. The functional balance of these two neuropeptides may be crucial in central control of reproduction in zebrafish.
